# Increased CO_2_/N_2_ selectivity by stepwise fluorination in isoreticular ultramicroporous metal–organic frameworks†

**DOI:** 10.1039/d4sc01525h

**Published:** 2024-05-16

**Authors:** Tuo Di, Yukihiro Yoshida, Ken-ichi Otake, Susumu Kitagawa, Hiroshi Kitagawa

**Affiliations:** a Division of Chemistry, Graduate School of Science, Kyoto University Kitashirakawa-Oiwakecho, Sakyo-ku Kyoto 606-8502 Japan yoshiday@ssc.kuchem.kyoto-u.ac.jp kitagawa@kuchem.kyoto-u.ac.jp; b Institute for Integrated Cell-Material Sciences (iCeMS), Kyoto University Institute for Advanced Study, Kyoto University Yoshida Ushinomiya-cho, Sakyo-ku Kyoto 606-8501 Japan

## Abstract

Exploration of porous adsorbents with high CO_2_/N_2_ selectivity is of great significance for reducing CO_2_ content in the atmosphere. In this study, a series of isoreticular ultramicroporous fluorinated metal–organic frameworks (MOFs) were prepared to explore the benefits of fluorinated ultramicropores in improving CO_2_/N_2_ selectivity. Gas adsorption measurements revealed that the increase in the number of fluorine atoms in a ligand molecule leads to the increased CO_2_ uptakes and CO_2_/N_2_ selectivity. Theoretical calculations indicate that the interaction between the fluorine atoms and adsorbed CO_2_ molecules enhances the CO_2_-philicity, offering useful insight into the improvement of CO_2_/N_2_ selectivity in isoreticular frameworks.

## Abstract 2

A series of isoreticular ultramicroporous fluorinated MOFs were synthesized. Both experimental and simulation studies showed that increasing the fluorine content leads to a marked increase in CO_2_/N_2_ selectivity owing to the CF⋯C(CO_2_) interactions.

## Graphical abstract

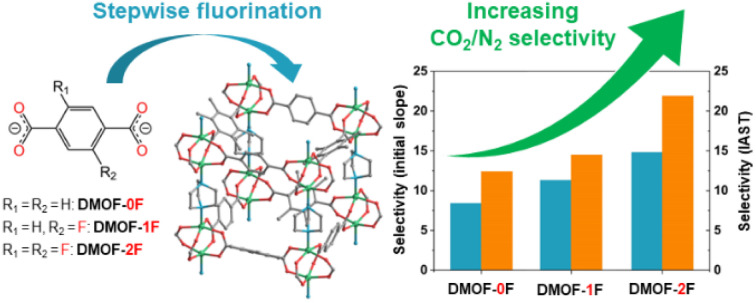

## Introduction

Excessive emissions of greenhouse gases, primarily carbon dioxide (CO_2_), can lead to the intensification of the greenhouse effect, resulting in global temperature imbalance.^1–4^ It is imperative to develop appropriate and effective methods to address the CO_2_ emitted from production and daily activities. Until now, carbon capture and sequestration (CCS) and direct air capture (DAC) technologies, which can efficiently capture CO_2_ from emission sources, are acknowledged as effective approaches for CO_2_ treatment.^5–8^ Exploration of efficient CO_2_ capture materials that meet the needs of above-mentioned scenarios is of utmost importance in reducing the impact of greenhouse effect. Although traditional inorganic adsorbents such as activated carbon and zeolite have shown prominent gas separation selectivities,^9^ their low structural designability has limited their use as CO_2_ capture adsorbents at low concentration in ambient air and indoor confined spaces.

Metal–organic frameworks (MOFs) have emerged as a new family of highly promising porous materials for CO_2_ capture because of their structural and chemical tunability.^10–12^ Given the self-assembling construction of metal ions/clusters and organic linkers *via* strong coordination bonds, MOFs have provided new avenues to accurately regulate both their surface and interior by judiciously selecting metal knots and organic ligands to regulate their CO_2_-philicity.^13,14^ It has been found that the interpenetration of microporous frameworks to produce ultramicropores (pore size: *ca.* 0.5 nm) leads to higher CO_2_ affinity, thus higher CO_2_/N_2_ selectivity, than those of the parent microporous MOF.^15^ In addition, chemical modifications, such as fluorination of component ligands, can also lead to increased CO_2_-philicity, because the favourable interactions between the quadrupole of CO_2_ and fluorine atoms can greatly enhance CO_2_ affinity.^16–19^ Thus, it is possible that the combination of fluorination with the ultramicroporous structure offers an optimal platform to systematically investigate the effect of fluorination on CO_2_/N_2_ selectivity. To this end, it is necessary to choose a series of isostructural MOFs with different degrees of fluorination, because uncertainties caused by different structural characteristics inevitably reduce the rationality of the obtained results. It should be mentioned that the fluorination of ligands greatly affects the framework structure in many cases,^20–22^ which will hamper our research to examine the fluorination effect on the gas sorption properties.

Chun and co-workers reported a family of pillar-layered MOFs; Zn^II^-paddlewheel dimers coordinate with bis-bidentate terephthalate (BDC^2−^) linkers to construct two-dimensional (2D) square grids, which are connected by bis-monodentate 1,4-diazabicyclo[2.2.2]octane (DABCO) linkers.^23^ Of particular importance is that the three-dimensional (3D) framework structure renders the ultramicropores (pore size ∼0.6 nm) and even remains unchanged by replacing hydrogen atoms on BDC ligands with other functional groups such as methyl and halogen groups,^23–26^ which is inherently suitable for the present research.

In this study, we selected the ultramicroporous Zn_2_(BDC)(tmBDC)(DABCO) (tmBDC^2−^: tetramethylterephthalate) as a parent MOF because of its high stability against water vapour compared with its analogues.^27^ Crystallographic, optical, and sorption studies confirmed the successful substitution of BDC ligands with 2-fluoro-BDC (1FBDC^2−^) or 2,5-difluoro-BDC (2FBDC^2−^) ligands while maintaining the framework structure. Theoretical calculations based on sorption isotherms demonstrated that increasing the fluorine content leads to a pronounced increase in CO_2_/N_2_ selectivity under low CO_2_ concentration conditions, primarily driven by intermolecular interaction between the fluorine atoms and adsorbed CO_2_ molecules. The present work would provide deeper insights into the rational design of ultramicroporous MOFs for controlling CO_2_/N_2_ selectivity.

## Methods

### Materials and chemicals

Zinc(ii) nitrate hexahydrate (Zn(NO_3_)_2_·6H_2_O; 99%), hydrochloric acid (HCl; 35–37 wt%), potassium permanganate (KMnO_4_; 99%), and *N*,*N*-dimethylformamide (DMF; super dehydrated) were used as purchased from Fujifilm Wako Pure Chemical Industries, Ltd. Terephthalic acid (H_2_BDC; 99%) and 1,4-diazabicyclo[2.2.2]octane (DABCO; 98%) were obtained from Tokyo Chemical Industry Co., Ltd. 2,3,5,6-Tetramethylterephthalic acid (H_2_tmBDC, 95%) and 3-fluoro-4-methylbenzoic acid (98%) were acquired from Combi-Blocks Inc. 2,5-Difluoroterephthalic acid (H_2_2FBDC, 95%) was obtained from Enamine Ltd. All these chemicals were used without purification. 2-Fluororoterephthalic acid (H_2_1FBDC) was synthesized according to the reported literature^28^ with modest modifications (ESI and Fig. S1†).

### Syntheses

#### Zn_2_(BDC)(tmBDC)(DABCO) (DMOF-0F)

DMOF-0F was synthesized according to the reported literature^23^ with minor modifications. Typically, Zn(NO_3_)_2_·6H_2_O (0.95 g, 3.2 mmol), H_2_BDC (0.27 g, 1.6 mmol), H_2_tmBDC (0.36 g, 1.6 mmol), and DABCO (0.18 g, 1.6 mmol) were mixed in 48 mL DMF. After sonication for 5 min, the mixture was stirred at room temperature for 3 h before being filtered to yield a transparent solution. Then, the solution was transferred into a Teflon-lined stainless-steel autoclave and heated at 110 °C for 48 h. After being cooled to room temperature, the transparent precipitate was collected and washed with DMF before vacuum drying at 120 °C overnight for further characterization. Yield: 0.47 g (47%). Elemental analysis calcd (%) for Zn_2_(BDC)(tmBDC)(DABCO): C, 49.78; H, 4.50; N, 4.47. Found (%): C, 49.78; H, 4.43; N, 4.55.

#### Zn_2_(1FBDC)(tmBDC)(DABCO) (DMOF-1F)

DMOF-1F was prepared by the procedure described above for DMOF-0F except that H_2_1FBDC was used instead of H_2_BDC. Yield: 0.52 g (50%). Elemental analysis calcd (%) for Zn_2_(1FBDC)(tmBDC)(DABCO): C, 48.39; H, 4.22; N, 4.34. Found (%): C, 47.51; H, 4.12; N, 4.32.

#### Zn_2_(2FBDC)(tmBDC)(DABCO) (DMOF-2F)

DMOF-2F was prepared by the procedure described above for DMOF-0F except that H_2_2FBDC was used instead of H_2_BDC. Yield: 0.54 g (51%) and the activation was performed at 105 °C instead of 120 °C. Elemental analysis calcd (%) for Zn_2_(2FBDC)(tmBDC)(DABCO): C, 47.08; H, 3.95; N, 4.22. Found (%): C, 46.84; H, 4.02; N, 4.15.

### Characterization

Powder X-ray diffraction (PXRD) measurements were performed with a Rigaku MiniFlex600 with 2*θ* ranging from 5–50° using Cu Kα radiation (*λ* = 1.5418 Å). Fourier transform infrared (FT-IR) spectra were measured using a PerkinElmer Spectrum 100 spectrometer in attenuated total reflection (ATR) mode. Thermal stability was characterized by thermogravimetric analysis (TGA) on a Bruker TG-DTA2000SA at a heating rate of 5 K min^−1^ under N_2_ atmosphere. The N_2_ (77 K and 273 K) and CO_2_ (273 K and 298 K) gas adsorption and desorption measurements were performed using a BELSORP-max volumetric adsorption system. Before each measurement, the activated powder sample was activated at 343 K for 12 h. Ideal Adsorbed Solution Theory (IAST) selectivity simulation was performed based on a graphical user interface software, GraphIAST.^29^ Single-crystal X-ray diffraction (SCXRD) measurements were performed on a Rigaku XtaLAB PRO diffractometer equipped with a HyPix-6000HE detector with a graphite monochromatized Mo Kα radiation (*λ* = 0.71069 Å) at 100 K. A single crystal was cooled by a stream of cooled nitrogen gas. Breakthrough experiments were conducted on a BELCAT II-Cryo-10 apparatus. A mixture of CO_2_/N_2_ (15 : 85 v/v) was passed through a column packed with powdered samples (*ca.* 0.3 g) at 273 K. The samples were activated at 70 °C for 20–30 min prior to the experiments. Geometry optimizations of all CO_2_-adsorbed MOFs were carried out based on grand canonical Monte Carlo (GCMC) simulations using Materials Studio software (Accelrys) based on the universal force field (UFF).^30^

## Results and discussion

**Fig. 1:**
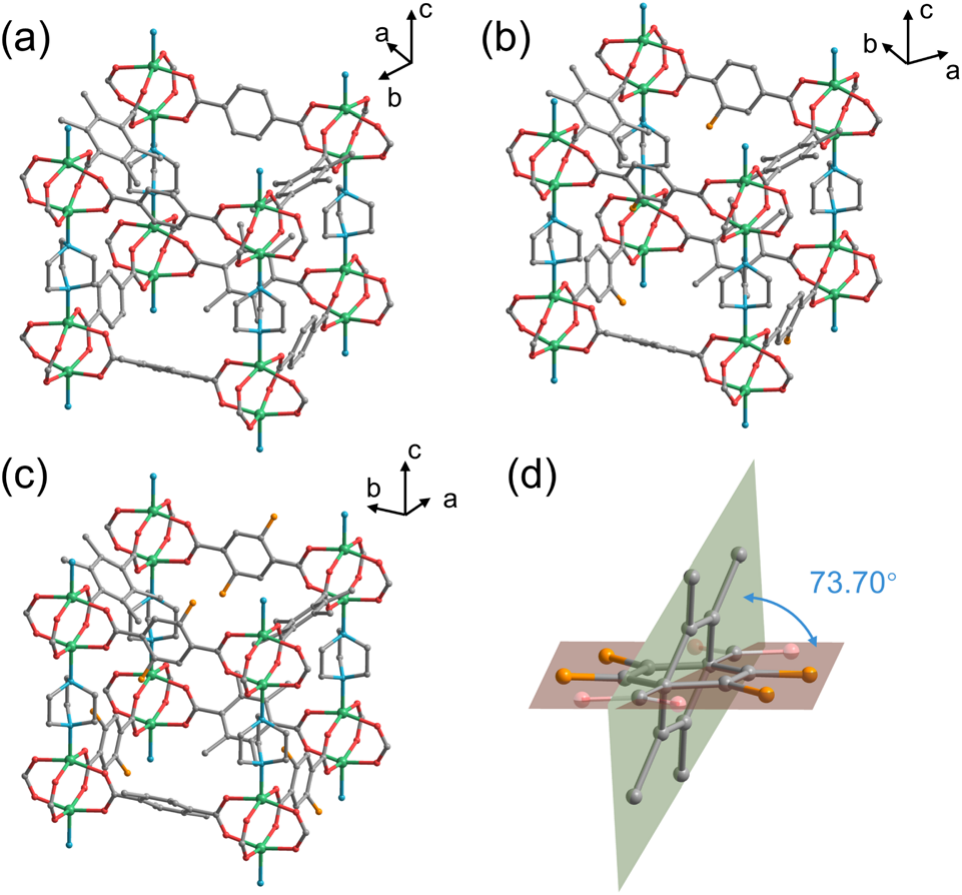
Expanded cubic-like structure of (a) DMOF-0F, (b) DMOF-1F, and (c) DMOF-2F, in which only one orientation of the disordered *x*FBDC ligand is shown. (d) Disorder of 1FBDC and tmBDC in DMOF-1F. Hydrogen atoms are omitted for clarity. Colour code: C: grey; F: orange; N: blue; O: red; Zn: green.

Crystal structures of the three MOFs were determined by SCXRD measurements, in which as-synthesized crystals were utilized for DMOF-0F and DMOF-1F, whereas heat-treated crystals (see below) were employed for DMOF-2F. Because the guest solvent molecules in the pore space could not be refined due to their disordered nature, the SQUEEZE function in PLATON software^31^ was utilized to remove the residual electron density. The PLATON/SQUEEZE procedure estimated a residual electron count of 175 e^−^, corresponding to 1.1 DMF molecules per Zn_2_(1FBDC)(tmBDC)(dabco) unit. Here, we elaborate only on the refined structures of two fluorinated MOFs, DMOF-1F and DMOF-2F, since the structure of the non-fluorinated MOF (*i.e.*, DMOF-0F) has already been reported in the literature.^23^ In fact, the fluorinated MOFs are isomorphous with the reported DMOF-0F. As shown in Fig. 1a–c, 2D (two-dimensional) square grids were constructed by bridging Zn dimers with bis-bidentate BDC-type ligands in a square-planar geometry in the *ab* plane. The paddlewheel units are connected by nitrogen atoms of DABCO molecules along the *c* axis, thereby expanding the planar network into a 3D structure. However, their space groups are not identical; namely, DMOF-1F belongs to the *I*4/*mcm* space group, whereas DMOF-2F crystallizes in the *P*4/*mmm* space group as in DMOF-0F.^23^ In the crystals, *x*FBDC (*x* = 1 or 2) and tmBDC ligands coexist in a disordered manner, where they have different orientations with a dihedral angle of 73.70° for *x* = 1, and 50.67° for *x* = 2 (Fig. 1d). The equivalent occupancy between *x*FBDC and tmBDC of 0.5 is consistent with the elemental analysis data. In addition, considering the fact that the *x*FBDC ligands can coordinate to Zn ions in a random manner (Fig. S2 and S3†), fluorine atoms in *x*FBDC ligands are disordered over four sites with an equivalent occupancy of 0.25 for DMOF-1F (Fig. 1b) and over two orientations, *i.e.*, 2,5- and 3,6-positions, with an equivalent occupancy of 0.5 for DMOF-2F (Fig. 1c). All three MOFs possess large 3D inter-connected voids with one-dimensional channels running along the *c* axis with windows through the *a* and *b* axes. Although the fluorine atoms slightly protrude into the channels, there are very little difference in the sizes of pores and windows, with an average diameter of *ca* 0.6 nm in all three MOFs. The procedure to estimate the pore sizes is shown in Fig. S4.† It is noteworthy that these pores are apparently larger than the kinetic diameter of common gas molecules (*e.g.*, N_2_ (0.364 nm) and CO_2_ (0.330 nm)), which allows their practical application in gas transportation and storage. In addition, it is expected that the surface density of fluorine atoms in the pores has a significant effect on the affinity for various gas molecules,^32,33^ which would be manifested in the adsorption isotherms, as discussed below.

**Fig. 2:**
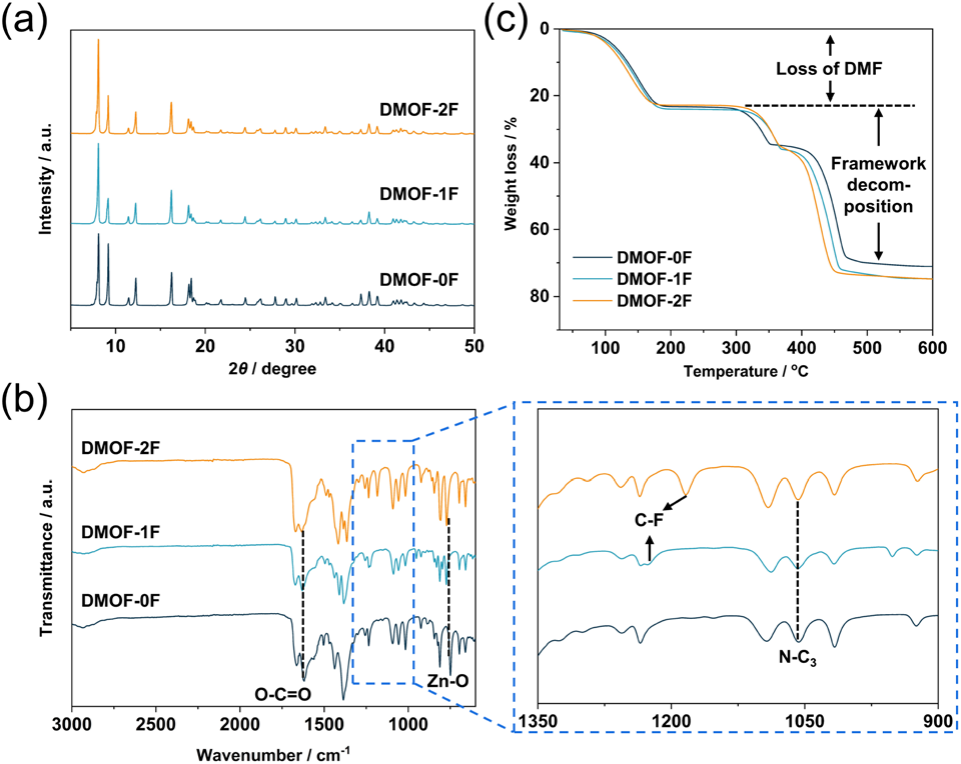
(a) PXRD patterns, (b) FT-IR spectra, and (c) TGA profiles of as-synthesized DMOF-0F (dark green), DMOF-1F (pale blue), and DMOF-2F (orange). In (b), enlarged spectra in the range of 900–1350 cm^–1^ are shown in the panel to the right.

PXRD measurement was utilized to confirm the phase purity of the polycrystalline samples. As shown in Fig. 2a and S5,† the PXRD patterns of the three as-synthesized MOFs match well with those simulated from the SCXRD data without any trace impurity peaks, indicating the high purity of the polycrystalline samples. FT-IR spectroscopy was applied to investigate the chemical structure of the as-synthesized MOFs. In Fig. 2b, the peak at 749 cm^−1^ can be assigned to the stretching vibration of Zn–O bonds originating from the dinuclear paddlewheel units.^34^ The peaks at 1619 is ascribed to the stretching vibration of carboxylate groups,^35^ whereas the peak at 1057 cm^−1^ is due to the asymmetric stretching vibration of N–C_3_ bonds in pillar DABCO molecules.^34^ In addition, the C–F stretching band was observed at 1227 cm^−1^ (DMOF-1F) and 1184 cm^−1^ (DMOF-2F) in fluorinated MOFs. The observation of conspicuous characteristic peaks of DMF, which was used as the reaction solvent, at 1664 cm^−1^ (C=O stretching), 1256 cm^−1^ (C–N stretching), and 660 cm^−1^ (O=C–N bending) indicates the inclusion of DMF in the as-synthesized MOFs.^36^ Thermal stability of the as-synthesized MOFs was evaluated using TGA in the temperature range from room temperature to 600 °C under N_2_ atmosphere. As shown in Fig. 2c, all the TG profiles exhibit similar thermal behaviour, showing a huge weight loss below 200 °C. Combining the results from FT-IR, we conclude that the decrease in weight is primarily caused by the removal of solvent DMF molecules adsorbed in the channel and on the surface of particles. Subsequently, there is a plateau between 200 and 300 °C followed by a weight loss possibly due to the removal of terephthalate or DABCO molecules. Based on the TG profiles, it can be concluded that the three MOFs are thermally stable up to approximately 300 °C under N_2_ atmosphere, which is comparable to those of some well-known MOFs; *e.g.*, HKUST-1 (Cu_3_(BTC)_2_; H_3_BTC: 1,3,5-benzenetricarboxylic acid; up to *ca.* 240 °C)^37^ and MOF-801 (Zr_6_O_4_(OH)_4_(fumarate)_6_; up to *ca.* 260 °C).^38^

Because the DMF molecules occupy the pore space throughout the frameworks, appropriate methods should be applied to completely remove the solvent species in the pores before subsequent adsorption measurements. After plenty of attempts, we finally determined that the dynamic vacuum at elevated temperature is the optimal method to achieve the complete removal of DMF in the pores while keeping the framework structure. All the activated MOFs after thermal treatment at 120 °C for DMOF-0F and DMOF-1F and 105 °C for DMOF-2F for 10–12 h exhibit the PXRD patterns similar to those of as-synthesized ones with respect to the peak positions and widths (Fig. S6†). Given that the vibrational bands of DMF molecules (1664, 1256, and 660 cm^−1^) and weight loss due to DMF evaporation disappear in the FT-IR spectra (Fig. S7†) and TG profiles (Fig. S8†), respectively, it is obvious that DMF molecules are completely removed through the dynamic vacuum, and therefore, the activated MOFs are available for gas adsorption measurements.

**Fig. 3:**
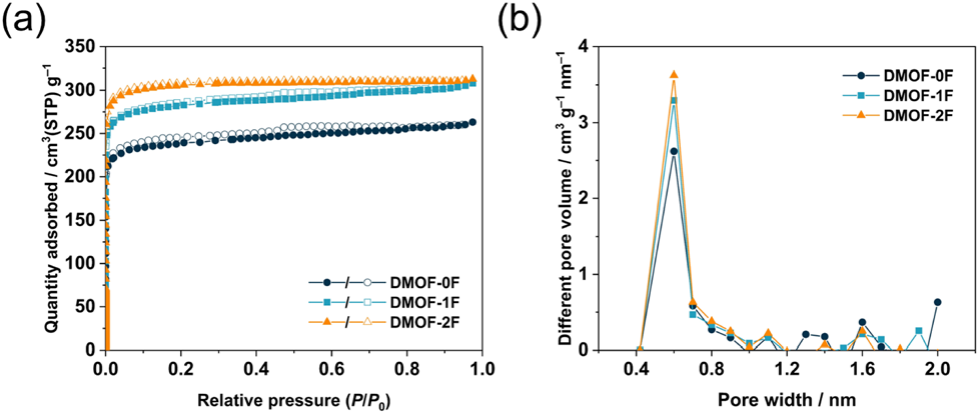
(a) N_2_ adsorption (closed symbols) and desorption (open symbols) isotherms at 77 K and (b) pore size distribution of DMOF-0F (dark green circles), DMOF-1F (pale blue squares), and DMOF-2F (orange triangles).

Porous properties of the three MOFs were evaluated by N_2_ adsorption/desorption measurements at 77 K. As shown in Fig. 3a, the three MOFs exhibit a steep increase in the low-pressure region (*P*/*P*_0_ < 0.01), suggesting the presence of abundant permanent micropores throughout the frameworks. Subsequently, they exhibit negligible N_2_ uptakes in the relative pressure range 0.1–0.8 with a typical type-I isotherm in the IUPAC classification.^39^ In the high-pressure region (*P*/*P*_0_ > 0.9), all three isotherms show a slight increase, indicating the presence of macropores arising from the inter-particulate voids due to the loose packing of particles. The uptake of N_2_ adsorption at *P*/*P*_0_ = 0.97 in DMOF-0F (263 cm^3^ g^−1^) is lower than those of DMOF-1F (308 cm^3^ g^−1^) and DMOF-2F (313 cm^3^ g^−1^). The Brunauer–Emmett–Teller (BET) surface areas of DMOF-0F, 1F, and 2F are estimated to be 949, 1123, and 1225 m^2^ g^−1^ (Table 1), respectively, which are close to the reported value for DMOF-0F (1100 m^2^ g^−1^).^23^ The total pore volumes (*V*_total_) calculated at a *P*/*P*_0_ of 0.97 are 0.41, 0.48, and 0.48 cm^3^ g^−1^, respectively. Pore size distribution calculated using the micropore analysis (MP) method shows that the three MOFs possess prominent ultramicropores with a size of *ca.* 0.6 nm (Fig. 3b), which is consistent with that expected from the crystallographic data (*ca.* 0.6 nm).

**Table 1:** BET surface area (*S*_BET_), total pore volume (*V*_total_), isosteric heat of adsorption (*Q*_st_), and CO_2_/N_2_ selectivity

	*S*_BET_ a (m^2^ g^−1^)	*V*_total_ b (cm^3^ g^−1^)	*Q*_st_ (kJ mol^−1^)	Selectivity (initial slope)	Selectivity^c^ (IAST)
DMOF-0F	949	0.41	19.3	8.4	12.4
DMOF-1F	1123	0.48	20.2	11.3	14.5
DMOF-2F	1225	0.48	23.3	14.8	21.9

a BET surface area.

b Total pore volume.

c Values at 0.3 bar.

**Fig. 4:**
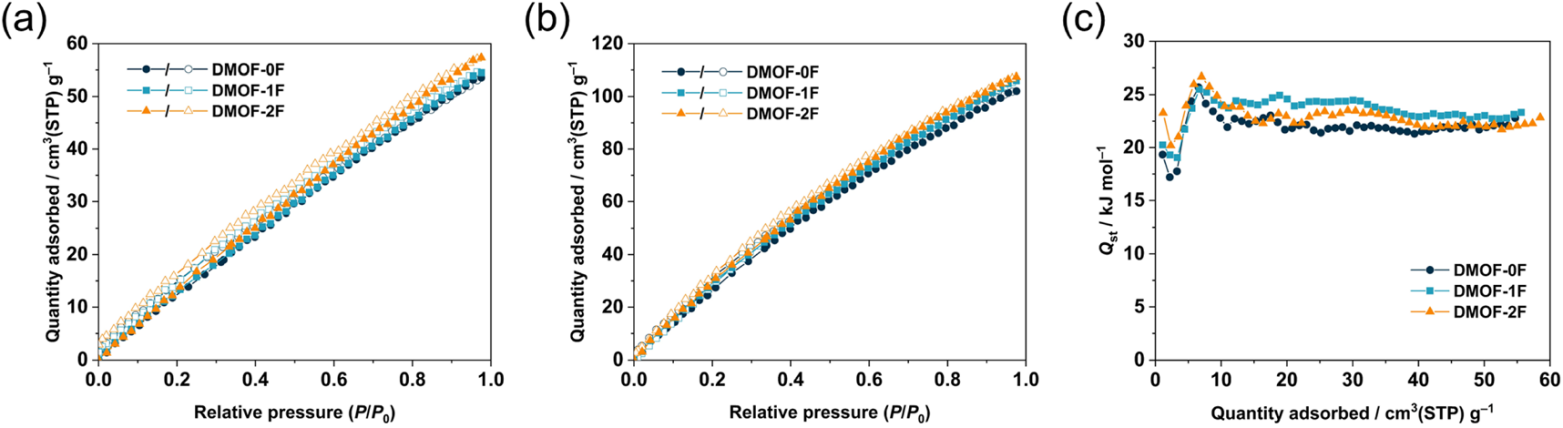
CO_2_ adsorption (closed symbols) and desorption (open symbols) isotherms of DMOF-0F (dark green circles), DMOF-1F (pale blue squares), and DMOF-2F (orange triangles) at (a) 298 K and (b) 273 K. (c) Isosteric heats of adsorption (*Q*_st_) for CO_2_ calculated from the data at 298 and 273 K.

Considering the relatively high BET surface areas (*S*_BET_), the present MOFs with comparable ultramicropore sizes with different fluorination environments allow us to investigate the effect of fluorination on CO_2_-philicity, because several fluorinated MOFs have been reported to exhibit Coulomb interactions between the negatively-charged fluorine atoms and positively-charged carbon atom in CO_2_ molecules.^33^Fig. 4a and b display the CO_2_ sorption isotherms of the three MOFs collected at 298 and 273 K, respectively. At both temperatures, all the MOFs show a gradual increase in CO_2_ uptake over the whole pressure region with a minimal hysteresis loop, indicating the physisorption-dominant process. They exhibit a relatively high CO_2_ uptake, possibly as a consequence of their high *S*_BET_ and *V*_total_. The CO_2_ uptake at 273 K follows the order DMOF-0F (102.1 cm^3^ g^−1^, 4.55 mmol g^−1^, 20.0 wt%) < DMOF-1F (106.1 cm^3^ g^−1^, 4.73 mmol g^−1^, 20.8 wt%) < DMOF-2F (107.4 cm^3^ g^−1^, 4.79 mmol g^−1^, 21.1 wt%). Although the lower *V*_total_ value of DMOF-0F estimated from the N_2_ sorption isotherm at 77 K may lead to the lower CO_2_ uptake, it is apparent that the higher CO_2_ uptake in DMOF-2F than that of DMOF-1F with comparable *V*_total_ is attributed to the higher fluorine content. These values are comparable or superior to some well-known MOFs, such as SNU-4 (Zn_2_(abtc)(DMF)_2_; H_4_abtc: 3,3′,5,5′-azobenzenetetracarboxylic acid, 20.6 wt%, surface area 1460 m^2^ g^−1^),^40^ SNU-5′ (Cu_2_(abtc)(DMF)_2_; 19.2 wt%, surface area 1260 m^2^ g^−1^),^40^ Zn_2_(BTTB)(DMF)_2_ (H_4_BTTB: 1,2,4,5-tetrakis(4-carboxyphenyl)benzene; 19.7 wt%, surface area 1370 m^2^ g^−1^),^18^ and SNU-21 (Cu_2_(TCM)(H_2_O)_2_, H_4_TCM: tetrakis[4-(carboxyphenyl)oxamethyl]methane; 18.4 wt%, surface area 905 m^2^ g^−1^).^41^ The reversible isotherm suggests that the MOFs under study can release the adsorbed CO_2_ molecules without the need of any additional thermal energy, which is advantageous for the real-world CO_2_ capture and regeneration of porous materials.^42^ The PXRD patterns after the CO_2_ adsorption/desorption measurement (Fig. S9†) confirmed the retention of the structure during the sorption process.

To examine the thermodynamics of the CO_2_ adsorption, isosteric heat of adsorption (*Q*_st_) toward CO_2_ were calculated from the Clausius–Clapeyron equation:^43^ ln(*P*_2_/*P*_1_) = *Q*_st_ × (*T*_2_ − *T*_1_)/(*RT*_1_*T*_2_), where *P*_*n*_ and *T*_*n*_ (*n* = 1 or 2) denote the pressure and temperature values, respectively, for the *n*th isotherm, and *R* is the gas constant expressed in the appropriate unit (8.314 J K^−1^ mol^−1^), using the adsorption isotherms at 298 and 273 K. As shown in Fig. 4c, the *Q*_st_ values are estimated to be 17.2–25.7 kJ mol^−1^ for DMOF-0F, 19.1–25.5 kJ mol^−1^ for DMOF-1F, and 20.2–26.7 kJ mol^−1^ for DMOF-2F, depending on the degree of CO_2_ loading, which lie in the typical range for CO_2_ adsorption of MOFs.^9,44^ It is noteworthy that the *Q*_st_ values, which are lower than the energy of the chemical bonds, is again indicative of the physisorption process (<40 kJ mol^−1^),^45^ which facilitates the controlled release of CO_2_ gas as mentioned above.^42^

**Fig. 5:**
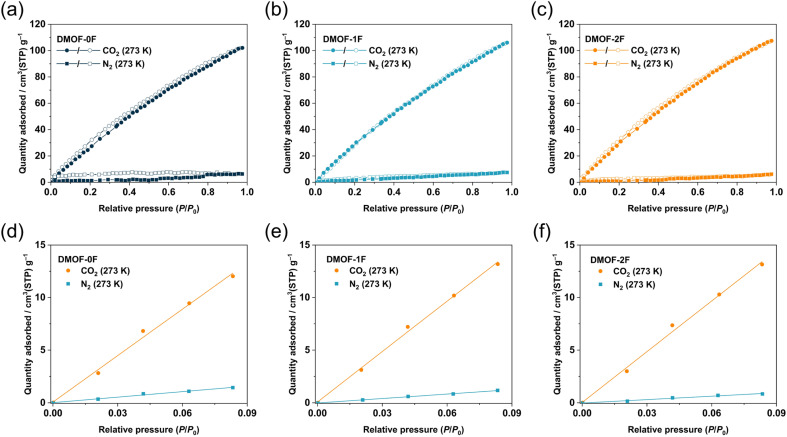
CO_2_ and N_2_ sorption isotherms of at 273 K for (a) DMOF-0F, (b) DMOF-1F, and (c) DMOF-2F. Initial slope fitting for CO_2_ and N_2_ adsorption isotherms at 273 K for (d) DMOF-0F, (e) DMOF-1F, and (f) DMOF-2F.

It is noteworthy that for the removal of CO_2_ from flue gas in coal power plants (also known as post-combustion CO_2_ capture), the CO_2_ concentration must be reduced to a certain level with respect to N_2_. Thus, it is necessary to explore adsorbents that exhibit a high CO_2_/N_2_ selectivity to enable efficient capture of CO_2_ under such conditions. The CO_2_ and N_2_ adsorption isotherms of the MOFs under study were compared to investigate the adsorption selectivity of CO_2_ over N_2_. As shown in Fig. 5a–c, the adsorption amount of CO_2_ is remarkably higher than that of N_2_ at 273 K and the CO_2_/N_2_ selectivity is estimated to be 8.4, 11.3, and 14.8 for DMOF-0F, DMOF-1F, and DMOF-2F, respectively, based on the initial slope method (Fig. 5d–f).^42^ Although the selectivity values are less than those of a certain number of MOFs reported so far,^1^ the results deliver the message that fluorination has a favourable effect on CO_2_/N_2_ selectivity. Dynamic breakthrough measurements using a gas mixture of CO_2_/N_2_ (15 : 85) at 273 K demonstrate the effective separation *via* a one-step experiment, although no significant differences were found among the three MOFs (Fig. S10†). The difference in CO_2_/N_2_ selectivity depending on the number of fluorine atoms (8.4–14.8) is more pronounced compared with that of ultramicroporous fluorinated MOF, Ni(tpt)(*x*F-OPA)(H_2_O) (tpt: 2,4,6-tri(4-pyridinyl)-1,3,5-triazine; H_2_OPA: *o*-phthalic acid; 13.8–15.8), with 1D channels of *ca.* 0.62 nm in diameter, in which 4-fluoro-OPA (*x* = 1; TKL-105), 3,6-difluoro-OPA (*x* = 2; TKL-106), and 3,4,5,6-tetrafluoro-OPA (*x* = 4; TKL-107) were utilized as a bridging ligand.^46^ Although the factors that determine the selectivity remain unclear in these systems, the CH⋯F hydrogen bonds between the pyridine moieties of and fluorine atoms of *x*F-OPA within the pore walls in TKL-106 and 107 would suppress the intermolecular CF⋯C(CO_2_) interactions between the *x*F-OPA ligands and CO_2_ molecules. Although DMOF-1F and DMOF-2F also involve CH⋯F hydrogen bonds, the C–H groups are in the fast-rotating DABCO ligands^47^ which may allow the effective intermolecular CF⋯C(CO_2_) interactions between *x*FBDC ligands and CO_2_ molecules. Park and co-workers reported the increased CO_2_/N_2_ selectivity by the partial substitution of 2-amino-BDC (NH_2_BDC^2−^) with 2,3,5,6-tetrafluoro-BDC (4FBDC^2−^) in amino-functionalized MIL-101(Cr) with a chemical composition of Cr_3_O(OH)(H_2_O)_2_(NH_2_BDC)_3_;^48^ the CO_2_/N_2_ selectivity in the 4FBDC-introduced MOFs (92–108) is significantly higher than that of the pristine MOF (51). However, the authors concluded that the increased CO_2_ adsorption in 4FBDC-introduced MOFs arises mainly from the decreased pore sizes (1.7 to 0.9 cm^3^ g^−1^) rather than the increased fluorine content. On the other hand, we succeeded in improving the CO_2_/N_2_ selectivity mainly by controlling the number of fluorine atoms in a BDC ligand while properly keeping the porous properties (0.41 to 0.48 cm^3^ g^−1^). Thus, the present result is the first demonstration of fluorinated ultramicroporous MOFs to control the CO_2_/N_2_ selectivity at a significant level by controlling the fluorine content (Table S1†).

**Fig. 6:**
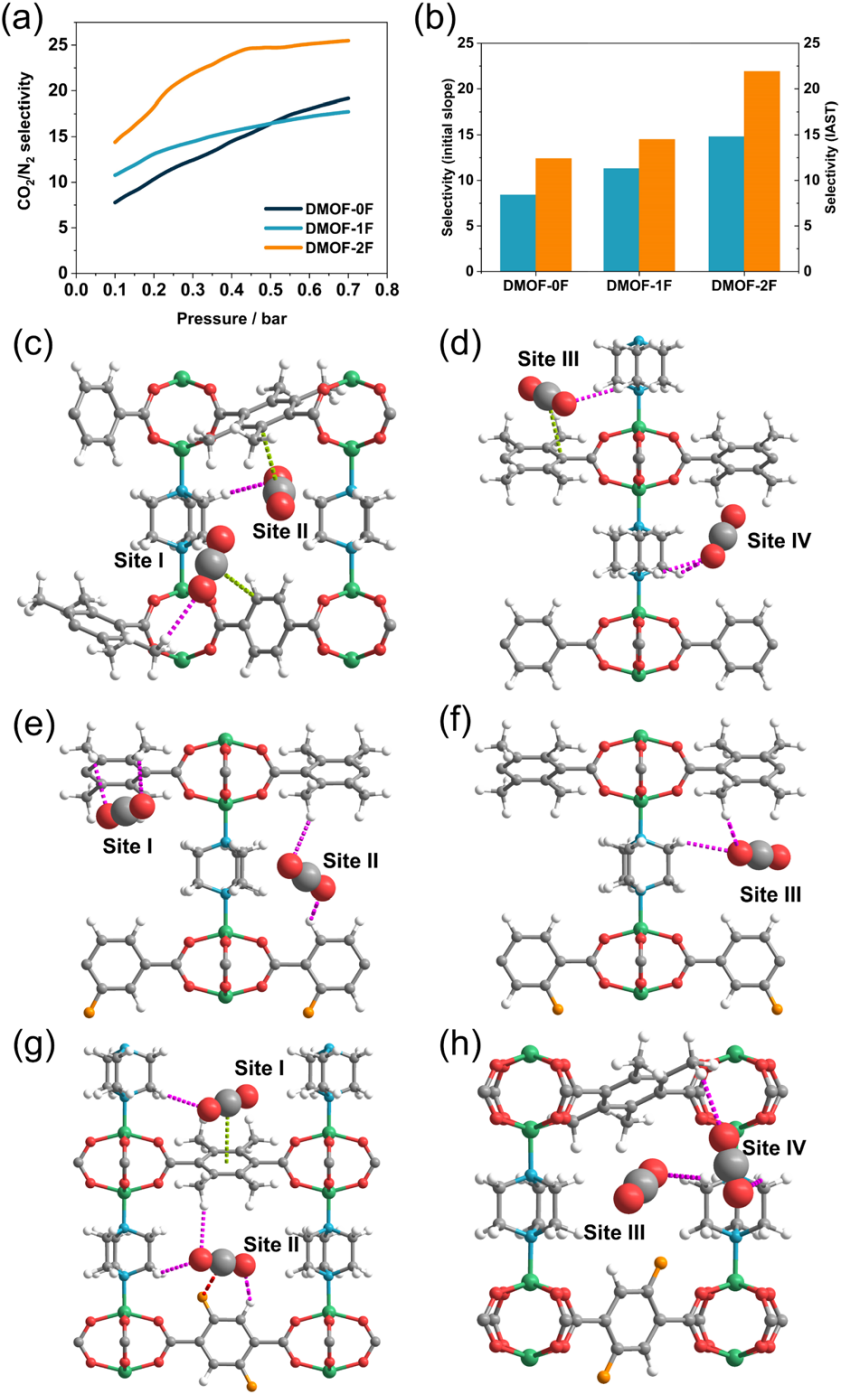
(a) Simulated IAST selectivity of CO_2_/N_2_ for DMOF-0F (dark green line), DMOF-1F (pale blue line), and DMOF-2F (orange line) under atmospheric CO_2_ concentration (*i.e.*, 500 ppm of CO_2_ to N_2_). (b) CO_2_/N_2_ selectivity of three MOFs based on initial slope method (pale blue) and IAST method (orange). Simulated interactions between the framework and adsorbed CO_2_ molecules in (c and d) DMOF-0F, (e and f) DMOF-1F, (g and h) DMOF-2F, where CO_2_ molecules with no effective interactions are emitted. CH⋯O(CO_2_), CF⋯C(CO_2_), and π⋯C(CO_2_) interactions are shown in pink, red, and light green dotted lines, respectively.

To demonstrate the ability of the three MOFs to capture trace CO_2_, their CO_2_/N_2_ selectivity at atmospheric concentration (500 ppm for CO_2_) was calculated using IAST.^29^ As illustrated in Fig. 6a, the CO_2_/N_2_ selectivity varies depending on the fluorination and increases with increasing pressure. The selectivity in the low-pressure region (<0.3 bar) is in the order DMOF-0F (12.4) < DMOF-1F (14.5) < DMOF-2F (21.9), which is consistent with the results obtained from the initial slope method (Fig. 6b). The difference between DMOF-0F and DMOF-1F (2.1) is less pronounced than that between DMOF-1F and DMOF-2F (7.4), which is consistent with the calculated *Q*_st_ values (Table 1).

GCMC simulations were conducted to elucidate the origin of the CO_2_ capture behaviour at the microscopic level. The geometric structures of the MOFs were optimized with the assumption that there are four CO_2_ molecules in each 1D channel in the unit cell at *P*/*P*_0_ = 0.3 at 273 K. In DMOF-0F, there are several CH⋯O(CO_2_) hydrogen bonding interactions between the tmBDC or DABCO ligands and CO_2_ molecules with an H⋯O distance of 2.88 Å (Site I), 2.89 Å (Site II), 2.94 Å (Site III), and 2.97 and 3.01 Å (Site IV) (*vs.* the sum of the van der Waals radii: 2.72 Å^49^) as shown in Fig. 6c, d and Table S2.† Such interactions were found in CO_2_-incorporated DMOF-1F framework, with a distance of 3.02 and 3.04 Å (Site I), 2.98 and 2.98 Å (Site II), and 2.90 and 3.00 Å (Site III) (Fig. 6e and f). In addition, DMOF-0F shows several π⋯C(CO_2_) interactions^19,50^ between CO_2_ molecules and benzene rings of BDC-based ligands, with the nearest C⋯C distance of 3.45 Å (Site I), 3.60 Å (Site II), and 3.53 Å (Site III). However, there is no significant interaction between the fluorine atoms and CO_2_ molecules in the pore of DMOF-1F, although a Coulomb attractive force is expected between the negatively-charged fluorine atoms and positively-charged carbon atom in CO_2_ molecules.^33^ We note that the increase in the number of CO_2_ molecules in a channel in the unit cell from 4 (corresponding to *P*/*P*_0_ ∼ 0.3) to 6 (corresponding to *P*/*P*_0_ ∼ 0.4) results in the formation of short CF⋯C(CO_2_) contacts with a distance of 3.30 Å (*vs.* the sum of the van der Waals radii: 3.17 Å;^49^ Fig. S11†). In DMOF-2F (Fig. 6g and h), CO_2_ molecules in the pores exhibit a significant interaction with fluorine atoms with an F⋯C distance of 3.33 Å (Site II in Fig. 6g). Such a favourable CF⋯C(CO_2_) interaction has also been found in fluorinated Ce-MIL-140 (CeO(4FBDC)),^51^ in which an CF⋯C(CO_2_) interaction with a distance of 3.3 Å results in a higher affinity for CO_2_ and boosts the CO_2_ selectivity. Therefore, it is apparent that the CF⋯C(CO_2_) interaction plays a pivotal role in the higher CO_2_/N_2_ selectivity in DMOF-2F than those of DMOF-0F and DMOF-1F.

## Conclusions

In this study, a series of isoreticular MOFs, namely, DMOF-0F, DMOF-1F, and DMOF-2F, possessing ultramicropores with varying degrees of fluorination were synthesized through the rational design of component species and framework structure. Both experimental and theoretical studies demonstrate that the CO_2_/N_2_ selectivity in the MOFs under study is increased by incorporating a higher number of fluorine atoms into the ultramicropore walls. It appears that the strong CF⋯C(CO_2_) interaction between 2FBDC ligands and CO_2_ molecules in DMOF-2F, predicted based on the Monte Carlo simulation, makes it the best adsorbent for CO_2_ among the three MOFs. Given that the study of the relationship between the fluorine content and CO_2_/N_2_ selectivity in ultramicroporous MOFs is still in its infancy, this work provides deeper insight into the design and synthesis of MOFs with controllable CO_2_/N_2_ selectivity.

## Data availability

Additional details regarding experimental and calculation data are given in the ESI.†

## Author contributions

T. D., Y. Y., and H. K. conceived the research and designed the experiments. T. D. performed all the experiments, measurements, and calculations. Dynamic breakthrough measurements were performed by T. D. with the support of K.-i. O. and S. K. T. D., Y. Y., and H. K. cowrote the manuscript. All the authors discussed and commented on the paper.

## Conflicts of interest

There are no conflicts to declare.

## Supplementary Material

SC-015-D4SC01525H-s001

SC-015-D4SC01525H-s002
