# Supplementary material for: Increased CO2/N2 selectivity by stepwise fluorination in isoreticular ultramicroporous metal–organic frameworks
Source: Chem Sci. 2024 May 16;15(25):9641–8. doi: 10.1039/d4sc01525h (PMC11205276; doi:10.1039/d4sc01525h)
Supplement: SC-015-D4SC01525H-s001 [file SC-015-D4SC01525H-s001.pdf]

Electronic Supplementary Material (ESI) for Chemical Science.
This journal is © The Royal Society of Chemistry 2024

Electronic Supporting Information (ESI)

Increased CO_2/N_2 selectivity by stepwise fluorination in isoreticular ultramicroporous metal-organic frameworks

Tuo Di,[a] Yukihiro Yoshida,[a] Ken-ichi Otake,[b] Susumu Kitagawa[b] and Hiroshi Kitagawa*[a]*

[a] Division of Chemistry, Graduate School of Science, Kyoto University, Kitashirakawa-Oiwakecho, Sakyo-ku, Kyoto 606-8502, Japan. E-mail: yoshiday@ssc.kuchem.kyoto-u.ac.jp; kitagawa@kuchem.kyoto-u.ac.jp

[b] Institute for Integrated Cell-Material Science (iCeMS), Kyoto University Institute for Advanced Study, Kyoto University, Yoshida Ushinomiya-cho, Sakyo-ku, Kyoto 606-8501, Japan

Synthesis of 2-fluoroterephthalic acid (H_21FBDC)

H_21FBDC was synthesized according to the reported literature[1] with minor modifications. Typically, 3-fluoro-4-methylbenzoic acid (2.04 g, 13.2 mmol) and $KMnO_4$ (6.85 g, 43.4 mmol) were added into 5% aqueous KOH solution (70 mL) and the mixture was refluxed at 110 °C for 4 h. The dark reddish-brown suspension was then allowed to cool to room temperature and stirred overnight. After filtration, the solution was diluted with water (50 mL) and acidified with conc. HCl to pH = 1. The white precipitate was collected by filtration, washed with water, and dried at 60 °C for 24 h to afford H_21FBDC as white powder (1.86 g, 77% yield). The ^1H NMR spectrum is shown in Fig. S1. Elemental analysis calcd (%) for $C_8H_5FO_4$: C, 52.19; H, 2.74; N, 0.00. Found (%): C, 52.03; H, 2.54; N, 0.00.

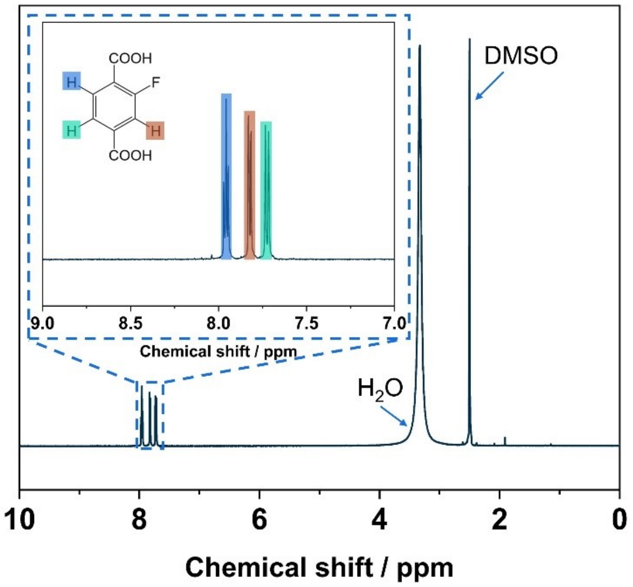

Fig. S1 ^1H NMR spectrum of H_21FBDC measured in DMSO-d_6.

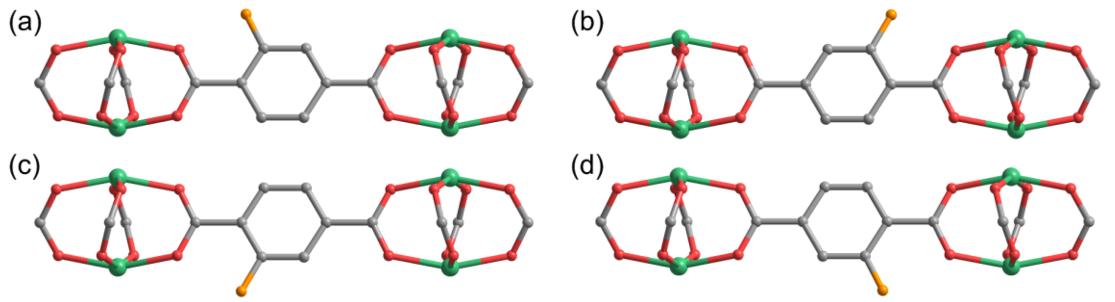

Fig. S2 Various modes of coordination of 1FBDC ligand between two paddlewheel-type Zn dimers. The occupancy factor of fluorine atom in 1FBDC linker is set to 1/3 of that of hydrogen atoms. Hydrogen atoms and DABCO pillars are omitted for clarity. Colour code: Zn: green; N: blue; O: red; C: grey; F: orange.

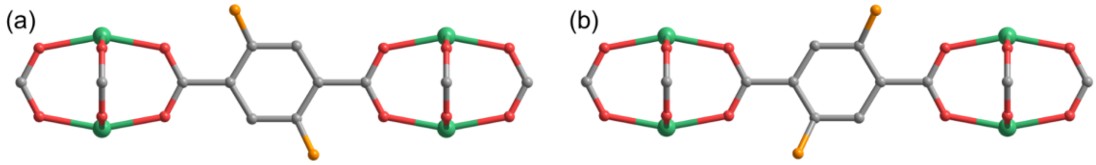

Fig. S3 Various modes of coordination of 2,5-difluoro-BDC (2FBDC) ligand between two paddlewheel-type Zn dimers. The occupancy factor of fluorine atoms in 2FBDC linker is the same as that of hydrogen atoms. Hydrogen atoms and DABCO pillars are omitted for clarity. Colour code: Zn: green; N: blue; O: red; C: grey; F: orange.

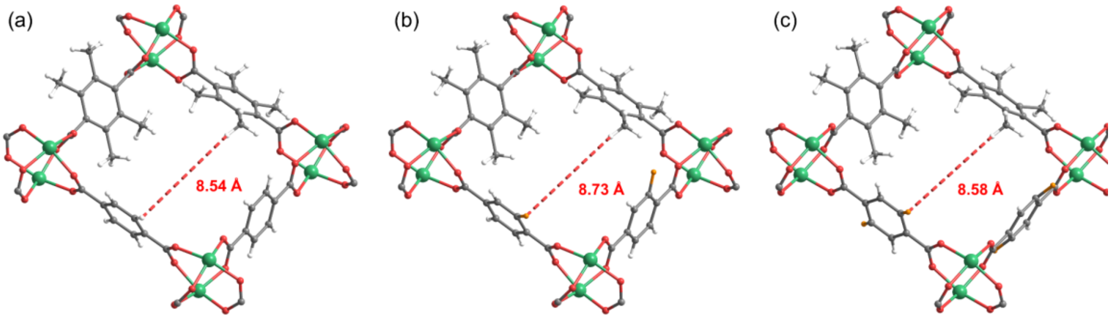

Fig. S4 Illustration of centre-to-centre distance between the closest atoms on an opposite side of 1D channel in (a) **DMOF-0F**, (b) **DMOF-1F**, and (c) **DMOF-2F**. Given that the molar ratio of *x*FBDC and tmBDC ligand is 1:1, we first measured the centre-to-centre distances between a fluorine atom of *x*FBDC (for x = 1 or 2) or a hydrogen atom of BDC (i.e., x = 0) and a hydrogen atom of a methyl group of tmBDC on an opposite side; 8.54 Å for **DMOF-0F** (x = 0), 8.73 Å for **DMOF-1F** (x = 1), and 8.58 Å for **DMOF-2F** (x = 2). Next, we subtracted the van der Waals radii of hydrogen and fluorine atoms, which are 1.20 and 1.47 Å, respectively,[2] from the above centre-to-centre distances. Therefore, it is apparent that the estimated pore sizes (6.14, 6.06, and 5.91 Å for **DMOF-0F**, **DMOF-1F**, and **DMOF-2F**, respectively) are comparable to each other and are in good agreement with the values estimated from the N_2 sorption measurements (ca. 6 Å; Fig. 3b).

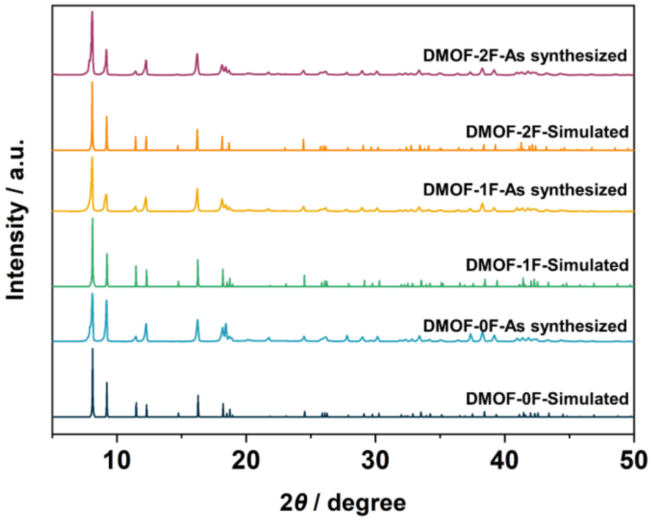

Fig. S5 Experimental and simulated PXRD patterns of polycrystalline **DMOF-0F**, **DMOF-1F**, and **DMOF-2F**.

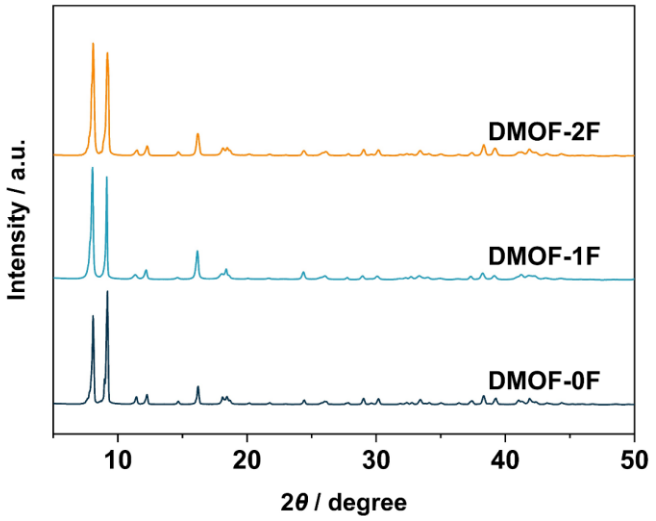

Fig. S6 PXRD patterns of polycrystalline **DMOF-0F** (dark green), **DMOF-1F** (pale blue), and **DMOF-2F** (orange) after activation.

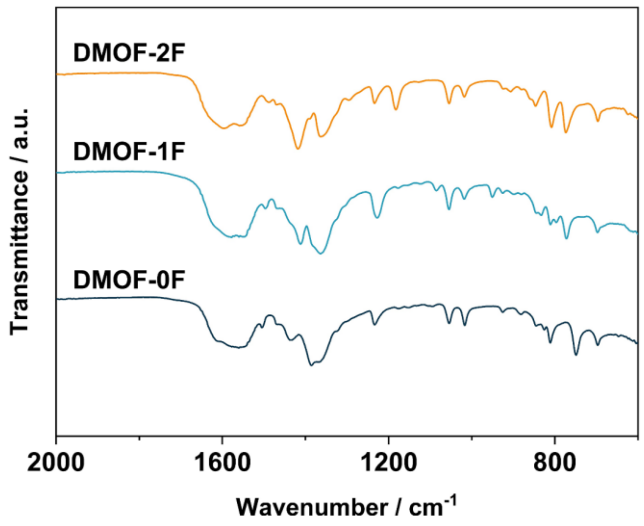

Fig. S7 FT-IR spectra of **DMOF-0F** (dark green), **DMOF-1F** (pale blue), and **DMOF-2F** (orange) after activation.

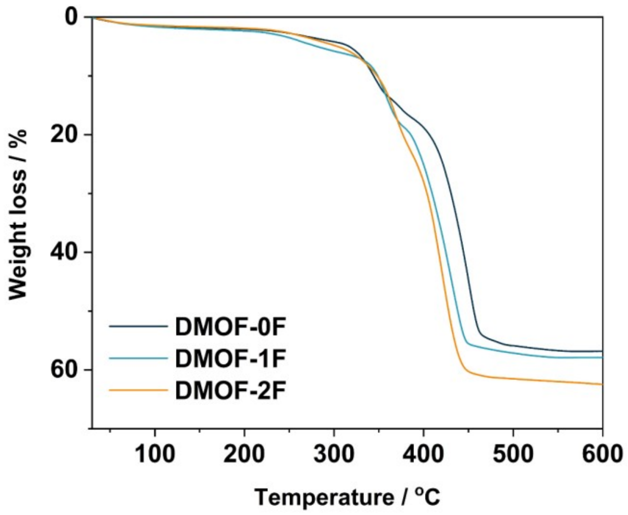

Fig. S8 TGA profiles of **DMOF-0F** (dark green), **DMOF-1F** (pale blue), and **DMOF-2F** (orange) after activation.

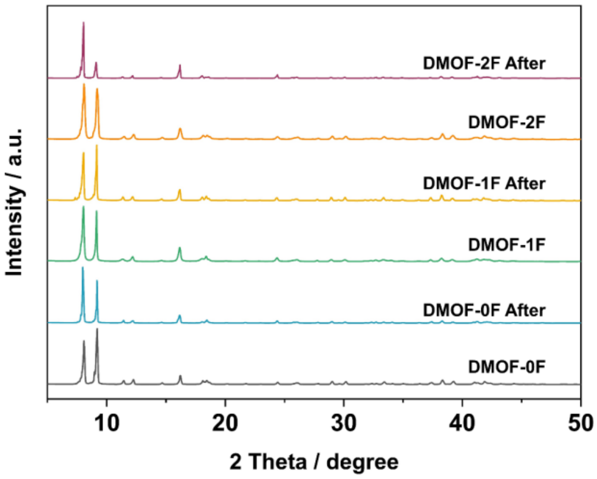

Fig. S9 PXRD patterns of samples after the CO_2 adsorption/desorption measurement together with those after activation (Fig. S6).

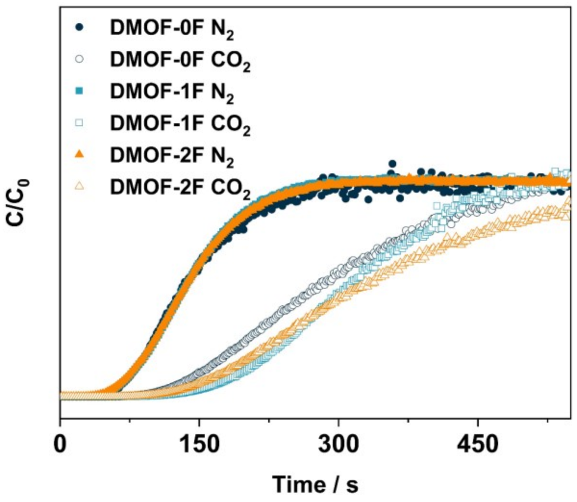

Fig. S10 Column breakthrough results of **DMOF-0F**, **DMOF-1F**, and **DMOF-2F** for CO_2/N_2 (15:85 v/v) at 273 K.

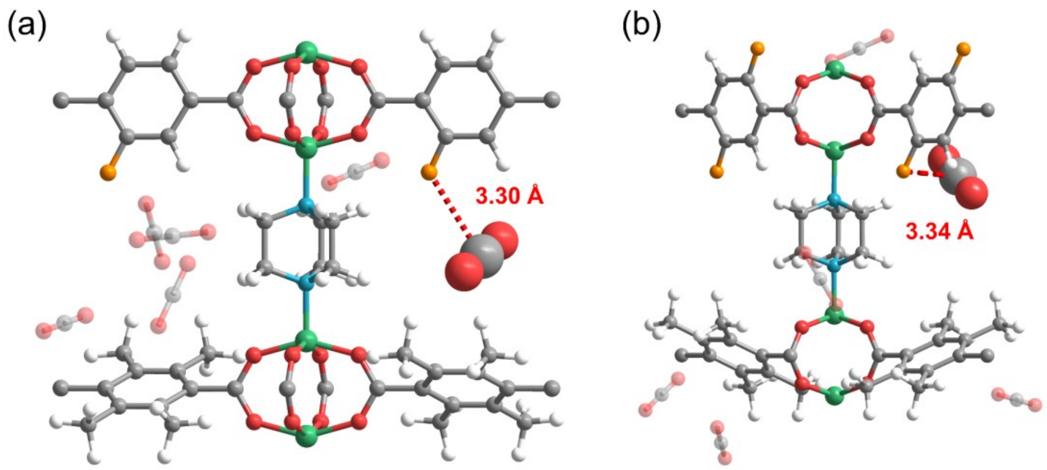

Fig. S11 Simulated interactions between the fluorine atom and adsorbed CO_2 molecule in (a) **DMOF-1F** and (b) **DMOF-2F** when there exist 6 CO_2 molecules in a channel in the unit cell. The CO_2 molecules forming a short CF···C(CO_2) contact (red dotted lines) are emphasized by enlarged representations.

Table S1 Summary of porosity and CO_2/N_2 selectivity in ultramicroporous fluorinated MOFs

Name	BET surface area / $m^2\ g^{-1}$	CO_2/N_2 selectivity		Ref.
		Initial slope method	IAST	
MOF-801	948	23	25	3
PF-MOF1	649	30	34	
PF-MOF2	626	95	41	
MIL-101(Cr)–NH_2	2909	–	51	4
MIL-101(Cr)–NH_2-$F_{0.5}$ (CrA-F0.5)	2385	–	108	
MIL-101(Cr)–NH_2-$F_{0.5}$ (CrA-F1)	968	–	92	
SIFSIX-2-Cu	3140	–	13.7	5
SIFSIX-2-Cu-i	735	–	140	
SIFSIX-3-Zn	250	–	1818	
TKL-105	1509	15.8	–	6
TKL-106	1636	15	–	
TKL-107	1454	13.8	–	
DMOF-0F	949	8.4	12.4	This work
DMOF-1F	1123	11.3	14.5	
DMOF-2F	1225	14.8	21.9	

Table S2 Simulated interaction types and interatomic distances in Fig. 6

MOF	Interaction type		Distance (Å)
**DMOF-0F**	CH···O(CO_2) **Site I**	H···O	2.88
	CH···O(CO_2) **Site II**	H···O	2.89
	CH···O(CO_2) **Site III**	H···O	2.94
	CH···O(CO_2) **Site IV**	H···O	2.97
	CH···O(CO_2) **Site IV**	H···O	3.01
	π···C(CO_2) **Site I**	C···C	3.45
	π···C(CO_2) **Site II**	C···C	3.60
	π···C(CO_2) **Site III**	C···C	3.53
**DMOF-1F**	CH···O(CO_2) **Site I**	H···O	3.02
	CH···O(CO_2) **Site I**	H···O	3.04
	CH···O(CO_2) **Site II**	H···O	2.98
	CH···O(CO_2) **Site II**	H···O	2.98
	CH···O(CO_2) **Site III**	H···O	2.90
	CH···O(CO_2) **Site III**	H···O	3.00
**DMOF-2F**	CH···O(CO_2) **Site I**	H···O	2.90
	CH···O(CO_2) **Site II**	H···O	2.85
	CH···O(CO_2) **Site II**	H···O	2.92
	CH···O(CO_2) **Site II**	H···O	2.99
	CH···O(CO_2) **Site III**	H···O	2.81
	CH···O(CO_2) **Site IV**	H···O	2.93
	CH···O(CO_2) **Site IV**	H···O	2.98
	CF···C(CO_2) **Site II**	C···F	3.30
	π···C(CO_2) **Site I**	C···C	3.41

References

1 S. T. Meek, J. J. Perry, S. L. Teich–McGoldrick, J. A. Greathouse and M. D. Allendorf, *Cryst. Growth Des.*, 2011, **11**, 4309–4312.

2 A. Bondi, *J. Phys. Chem.*, 1964, **68**, 441–451.

3 D. M. Venturi, M. S. Notari, R. Bondi, E. Mosconi, W. Kaiser, G. Mercuri, G. Giambastiani, A. Rossin, M. Taddei and F. Costantino, *ACS Appl. Mater. Interfaces*, 2022, **14**, 40801–40811.

4 J. M. Park, G.-Y. Cha, D. Jo, K. H. Cho, J. W. Yoon, Y. K. Hwang, S.-K. Lee and U.-H. Lee, *Chem. Eng. J.*, 2022, **444**, 136476.

5 P. Nugent, Y. Belmabkhout, S. D. Burd, A. J. Cairns, R. Luebke, K. Forrest, T. Pham, S. Ma, B. Space, L. Wojtas, M. Eddaoudi and M. J. Zaworotko, *Nature*, 2013, **495**, 80–84.

6 D.-S. Zhang, Z. Chang, Y.-F. Li, Z.-Y. Jiang, Z.-H. Xuan, Y.-H. Zhang, J.-R. Li, Q. Chen, T.-L. Hu and X.-H. Bu, *Sci. Rep.*, 2013, **3**, 3312.
